# Impetigo Herpetiformis: Review of Pathogenesis, Complication, and Treatment

**DOI:** 10.1155/2018/5801280

**Published:** 2018-04-04

**Authors:** Nastaran Namazi, Sahar Dadkhahfar

**Affiliations:** Skin Research Center, Shahid Beheshti University of Medical Sciences, Tehran, Iran

## Abstract

Impetigo herpetiformis (IH) is among rare dermatosis of pregnancy, which is currently considered as a form of generalized pustular psoriasis. It is diagnosed by characteristic lesions of erythematous patches and grouped pustules mostly in the third trimester of pregnancy and may have systemic associations. A variety of complications have been reported in the course of IH. Treatment of IH can be quite challenging, and a number of treatment options have been reported to be effective for the management.

## 1. Introduction

Impetigo herpetiformis (IH) is a rare dermatosis of pregnancy that can be life threatening. It is currently considered as a form of generalized pustular psoriasis despite the previous opinion that illustrated it to be a separate entity [1]. The condition mostly occurs in the third trimester of pregnancy and usually resolves after delivery; however, there is the possibility of recurrence in the following pregnancies [2].

## 2. Pathogenesis

The etiology of IH is yet to be elucidated [3]. According to some evidence such as the number of familial cases, genetic factors may influence the development of IH [4]. Based on the previous studies, most of the cases of generalized pustular psoriasis without history of psoriasis vulgaris carry homozygous or compound heterozygous mutation of Interleukin 36 Receptor Antagonist (IL36RN) that encodes IL-36 receptor antagonist [2].

IL36, which is not found in normal skin, is induced by other cytokines such as tumor necrosis factor-*α*, IL-17A, and IL-22 taking part in some pustular dermatoses [5]. Report of two cases with IH in Japan showed homozygous and heterozygous IL36RN mutations [6]. Recently, mutation of IL36RN has also been reported in a Chinese IH patient [7].

While the proportion of IL36RN negative patients who develop IH should be clarified, this mutation is considered to have promising role in predicting IH occurrence and preventing the possible risk to mother and fetus [6].

While it is difficult to consider a cause and effect role, there are certain conditions that are found to be associated with IH with the most recognized one being hypocalcemia. Conditions that have been reported to be the underlying cause of the hypocalcemia in patients with IH include hypoparathyroidism [8], hypoalbuminemia [9], low levels of vitamin D, and reduction in ionized serum calcium concentrations due to malabsorption. Hypoparathyroidism is the most prominent condition that is known to have a possible role in IH [8]. Although the mechanisms are poorly understood, several drugs might induce IH. In a report of IH triggered by N-butyl-scopolammonium bromide, IH developed in 34th week of gestation after five days of drug ingestion [10]. Ritodrine hydrochloride, a drug that has been used to suppress preterm uterine contractures, has also been reported to induce IH [11].

## 3. Clinical Features

**Figure 1:**
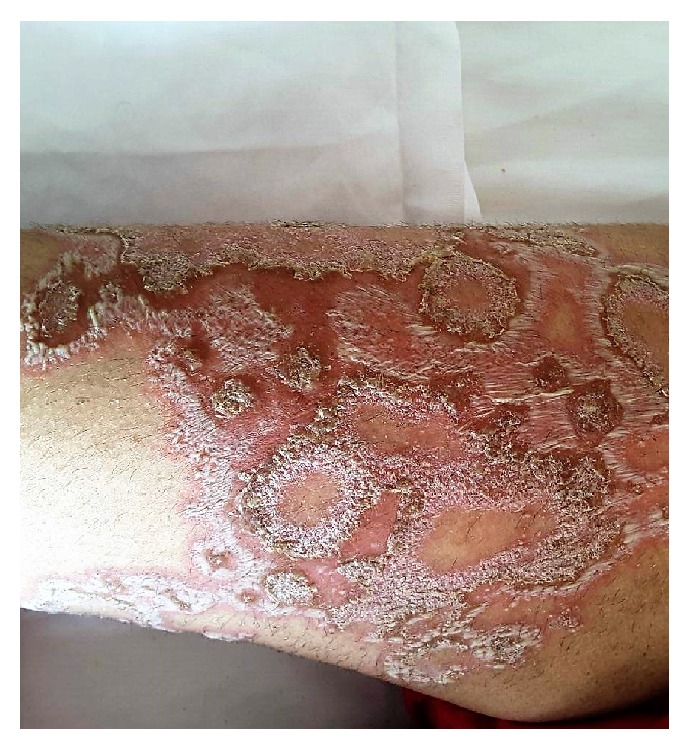
Annular lesions of pustular psoriasis of pregnancy on left thigh of a patient in the 36th week of the first pregnancy.

The characteristic lesions are erythematous patches with marginal grouped sterile pustules, primarily appearing in flexural regions, as they extend centrifugally, may develop erosion and crust, and may even become impetiginized (Figure 1).

Although not common, vegetative lesions similar to* Pemphigus vegetans* can be encountered in patients. Nail involvement and mucosal lesions in tongue, mouth, and even esophagus can be observed [8].

Hypoparathyroidism and hypocalcemia might be encountered in IH [12].

## 4. Complications

An important aspect of impetigo herpetiformis is the complications that might accompany IH or occur as a result of it endangering the life of mother and fetus [13]. A significant proportion of these complications are related to the placental insufficiency and electrolyte imbalance, on top of which is the alteration of serum calcium as mentioned above. Systemic symptoms such as nausea, vomiting, fever, chills, diarrhea, hypovolemic shock, seizure, and malaise [3] and laboratory findings such as leukocytosis, elevated ESR, hypocalcemia, hypoalbuminemia, and iron deficiency anemia may be associated with IH [14]. Gestational hypertension has been reported to complicate the condition of a woman diagnosed with IH in the 32nd week of pregnancy [15].

Additionally, IH has been associated with increased prenatal complications like intrauterine growth restriction as a result of placental insufficiency, premature rupture of membrane, and even stillbirth [16]. There is also a report of an infant being borne with Ondine's curse (central hypoventilation syndrome) from a mother with typical presentation of IH in her 8th month of pregnancy [17].

Recurrence has been described in up to nine pregnancies in a young woman. This was also precipitated by the intake of oral contraceptive pills (combination of ethinyl estradiol and progesterone) in the same patient [18].

## 5. Treatment

The main challenges are the critical condition of mother and fetus and the possible teratogenicity of the drugs that are being used in treatment of IH. Although many treatment options have been proposed for IH, there is no specific guideline and the evidence for efficacy of treatments is poor. Fluid and electrolytes imbalances especially hypovolemia, hypocalcemia, and low level of vitamin D should be *p* corrected promptly [19].

## 6. Corticosteroids

Systemic corticosteroids that have been historically used in the treatment of pustular psoriasis remain the mainstay of treatment. The initial dose in mild to moderate cases is 15–30 mg daily. If required, the dose can be increased to 40–60 mg and even up to 80 mg daily [19].

The main concern of corticosteroid therapy in pregnancy is the increased incidence of cleft palate [20]. Since IH commonly takes place late in the third trimester of pregnancy, corticosteroid therapy can be considered as a safe choice. Potent topical corticosteroid therapy may carry the risk of fetal growth restriction [21]. Therefore, it is reasonable to consider the use of mild to moderate corticosteroids rather than potent or very potent ones [22].

## 7. Cyclosporine

Cyclosporine is a therapeutic option in patients unresponsive to corticosteroids. According to literature, cyclosporine has been used in treatment of 14 patients with IH, most of them in combination with systemic corticosteroids. Cyclosporine was used at a dosage of 2–7.5 mg/kg/day with variable outcomes [23, 24]. After initiation of cyclosporine the corticosteroid can be tapered.

There is a report of complete clearance after treatment with cyclosporine initiated with a dose of 4 mg/kg followed by tapering of previously prescribed prednisolone in a week [25]. The last dose of cyclosporine was given 3 days before delivery followed by administration of high potency topical steroid.

Similar to any medication used during pregnancy, there are concerns about the safety of cyclosporine. Studies investigating the complications of cyclosporine mostly performed in renal transplant patients have shown no impairment of renal function and a weak likelihood of premature rupture of membranes [26]. In fact, placental transfer of cyclosporine seems to be dose dependent, and, with proper monitoring of fetus, it can be used harmlessly as an alternative for corticosteroids. The risk of maternal hypertension should be considered [27].

## 8. Antibiotics

The administration of antibiotics seems to be effective in IH, although antibiotics cannot control the disease entirely [28].

Antibiotic therapy can be considered in mild cases, and additive or alternative treatments such as corticosteroids can then be considered if antibiotics appear to be ineffective. Antibiotic therapy especially by cephalosporins is advised, despite the fact that the pustules are sterile [29]. Overall, cephalosporins are safe during pregnancy, but older cephalosporins are being preferred [27].

Ampicillin, macrolide, and clofazimine are among antibiotics that has shown efficacy in treatment of IH.

## 9. Biologic Agents

Anti TNF-*α* drugs such as infliximab and adalimumab are considered as pregnancy category B drugs. Although current data have not demonstrated an augmented risk in fetal complications in patients exposed to TNF-*α* blockers during pregnancy, their regular use during pregnancy is not approved by the United States Food and Drug Administration (FDA) [30].

According to the board of the National Psoriasis Foundation, infliximab is one of the best therapies for IH; however, this is contrary to the guideline of the European Academy of Dermatology and Venereology that does not advocate the use of adalimumab or infliximab during pregnancy. There are reports of refractory cases of psoriasis and IH treated by infliximab during pregnancy with favorable outcomes [31].

Ustekinumab has been reported to efficiently treat a case of recalcitrant severe pustular psoriasis during pregnancy with satisfactory result [32].

## 10. Phototherapy

NBUVB is considered a safe option during pregnancy and it can be added to therapy when there is not an adequate response to corticosteroids [33]. Although some studies have reported decreased folate levels during pregnancy due to NBUVB, it is not a major concern in the third trimester, the peak time of incidence of IH. However, folate deficiency in the first trimester may cause the development of neural tube defects [27].

PUVA is relatively safe and its administration has caused no increase in the risk of congenital malformations or infant mortality, but it may result in low birth weight infants [34].

## 11. Retinoids

Although, due to their teratogenic effects, all of the systemic retinoids are contraindicated during pregnancy, they have been used for IH treatment after the delivery. If administration of systemic retinoid is considered after the delivery, the informed consent for appropriate contraception should be taken from the mother [35].

## 12. Methotrexate

While methotrexate administration is not allowed during pregnancy, it has been used for treatment of IH successfully during puerperium [12].

## 13. Conclusion

Impetigo herpetiformis is a term that appears to be a misnomer, since it is neither caused by the bacterial pathogens nor caused by viral ones. It seems to be a variant of pustular psoriasis that has genetic, immunological, and biochemical milieu and may pose a major risk to both mother and fetus. Due to rarity of the disease, there is no controlled study or guideline for treatment. Many aspects of this disease is yet to be determined.

## Abbreviations

IH: Impetigo herpetiformis.

## Additional Points

Key Message. Impetigo herpetiformis is a life threatening dermatosis with risks to mother and fetus. Treatment of impetigo herpetiformis can be quite challenging, and a number of treatment options have been reported to be effective for the management with weak evidence.
